# A Doctor’s Responsibility

**Published:** 1877-01

**Authors:** Ben. H. Pratt


// THE BISTOURY.

Vol. XII. ELMIRA, N. Y., JANUARY, 1877. No. 4.

### A DOCTOR'S RESPONSIBILITY.

BEN. H. PRATT.

"Well, Doctor, how's business?"

"Dull—very dull!"

"That speaks well for the health of the city, does it not?"

"Well, yes; but it's not always safe to judge of the health of a place from the activity or otherwise, of its doctors."

"Ah! How do you explain that?"

"Why, you see, in this city people don't send for a doctor until they are sick, and don't retain him any longer than they really need him. So a doctor may suddenly be immersed in business for a week, and the next week may be absolutely destitute of a single patient. Where I came from it is quite different. In Philadelphia there are plenty of rich people addicted to big dinners, late suppers and large parties; where the men doff their heavy business suits and broad solid shoes for thin broadcloth and patent leather boots; and the ladies lay off their warm vests in order to appear in low necked dresses and no sleeves; and all indulge in ices while in a state of fever glow from excessive dancing in over-heated drawing rooms. The results are: Derangements of the stomach, indigestions, prostration of the whole system from undue stimulation, nervous difficulties of an acute character, arising from unseasonable indulgence and loss of sleep, together with a multitude of disorders occasioned by irregularities of diet and inadequate rest. Then come colds, neuralgias, rheumatism, catarrhs, milder forms of pulmonary complaints, headaches, and a long train of troubles which only need rest and quiet to complete a seemingly thorough restoration, but for which the afflicted must needs have a doctor, if it's only to find out what the trouble is, and to satisfy themselves that their complaints are not of the serious nature, of which the ailing are always inclined to believe themselves the victims. It is such trifling difficulties as these which are remunerative to the physician, and seem to entail none of those weighty responsibilities which a genuine case of sickness burdens the doctor with. He merely precribes some simple placebo, and advises rest and quiet, nature's remedies, which are the real medicines, though made to appear as secondary considerations and adjuncts to his prescriptions, which latter are always couched in formidable characters of cabalistic look, and in doctors' Latin which give them a wondrous significance, and over which the apothecary nearly explodes with laughter as he solemnly passes behind his prescription counter to compound. Why, between you and me, that's what those little cubby-holes in the drug shops are for—merely to conceal the smile of the druggist who alone knows the contents of the prescriptions—while he colors a vial of water, '*aquæ puræ*, ℥ ij,' with a little bit of cochineal, '*cocci pulv.* ℈ iss,' adds a few drops of rose water, '*aquæ rosæ*, gtt xx,' and tails it off with a 'mix it' written in such execrable Latin as '*fiat mistura.*' All of which the doctor prescribes gravely, the druggist dispenses solemnly, and the patient seizes and takes *secundem artem*, and with implicit faith. Of course the sick man or woman gets well, the doctor gets a good round fee, the druggist secures his divvy, and all is lovely, for we all live well and are happy. But here a man actually waits until he is sick—sick abed—wife has used all her remedies, old women have exhausted their stock of herbs and plasters and blisters and washes, and then, if he don't get any better, and is likely to die, they send for a doctor and he's got his hands full in a minute, and a fearful responsibility resting upon him to bring that man out of the clutches of death; in fact, perform a medical miracle or forever after have a murder chucked at his reputation whenever his name is suggested as a medical authority. That's why so many medical fools in our big cities get a big reputation as healers—they merely attend a patient who isn't really sick, and watch nature cure him; and that's the reason why so many really skillful country doctors can't earn a decent living; they are never called until disease has got such a hold of a man that all the doctors in creation can't save him, and the case turns on good or bad luck, as it happens to be. Now, you see how it is that the activity or idleness of the doctors is no criterion of the health of a place. Other matters must be considered, and other conditions must enter into the account. As a general rule, the wealthier a place is, the more sickness there appears to be, and where there's little or no wealth, the sick are more likely to die, because they are really sick, and not temporarily indisposed from indulgences and exposures; while in a town where all are comparatively poor, there isn't a show for a doctor to make a wood-chopper's wages. A surgeon may get an occasional job, but the doctor is usually the unhappiest looking man in the place."

The above is the purport of an interview between the writer and a physician of undoubted skill and acknowledged ability, in a provincial city in Pennsylvania, and it only confirmed, in the writer's mind, what he has long been very firmly impressed with, viz: that our city doctors live upon the follies of their patients, and build brown stone fronts, drive fast horses and become renowned, through the mania of upper-tendom to violate the laws of nature, and defy the immutable in order to gratify their vanity and their appetite.

The great increase exhibited of late years in the variety and phases of disease, is, without any doubt whatever, due to the follies of social life in the great cities. Intemperate eaters as well as intemperate drinkers; intemperate dressers and intemperate sleepers; intemperate dancers and intemperate idlers; all are gravitating to the same end, and will, in a few years, entail feebleness upon the whole nation. As the heart is the great vital center of the body human, so is the metropolis the foundation of health or disease to the great body social. What now are only temporary ailments will, by a gradual process of reducing the system, become chronic diseases and must be transmitted from generation to generation, until the normal man becomes a rare biped in nature. Can men and women, enfeebled by dissipations oft, leave to the world other than a legacy of puny offspring?

But are the devotees of society wholly to blame for this condition of things? Is it not a grievous sin of omission in a physician practicing among the giddy of the social world, to attend such cases as our medical friend mentions, and yet reveal by no sign whatever, the real causes of his patients' ailments? For what purpose are men made doctors? Is it merely to await the call of the hyperfashionable, administer their placebos, and take big fees for no equivalent rendered? Is he who does this better than the highwayman who stops the traveler on his journey and relieves him of his money?

Perhaps one may be laughed at for a fool, who condemns the practice which the great body of physicians admit to be legitimate and which brings large fees to their pockets. Perhaps, too, this source of wealth-getting is not any more to be criticised than that from which thousands of men are heaping up fortunes. The credulity of the great mass of men and women—their ignorance of everything pertaining to physiological, pathological and hygienic knowledge—the singular faith which one man puts in another because the latter has heard a few lectures upon medicine and has a latin diploma of some medical college, and which he can't read himself—the confidence in doctors entertained by the fashionable *ignorami*, who revel in all sorts of indulgences and feel no alarm, simply because they are assured in their own minds that their good doctor will be on hand the next morning to bring them around all right—the utter indifference which society exhibits to all the laws of health, and the risks it will take merely to secure the fleeting pleasures of the hour—these are what work such irremediable havoc in our national health and physique. But all this might be avoided if our doctors were only honest with their patients, and would tell the butterflies and peacocks of society that notwithstanding nature is always standing ready waiting to mend what folly has torn to pieces, every such effort of nature impairs, to a greater or less degree, her strength, and by and by there will be left nothing to draw from, and the victim must succumb to his or her wasted strength and become a hopeless invalid. Nature will stand a marvelous amount of abuse—does stand it, every day and hour even from those who think they are acting in obedience to her laws—but there is a point at which even she must give up, and declare her inability to lend further help. We see the results on every hand. Few live, now days, to the ripe age of three score and ten—every family, nearly, has its pale and helpless invalid who is paying the price of its own or its progenitor's violation of all hygienic laws—diseases of new and peculiar character are appearing, which baffle the skill of the physician and make one wonder what we are coming to—our friends who last year were the gayest of the gay, and whose ability to endure night after night of dissipative indulgence and come out as rosy and as cheery as a crowing infant from its crib at dawn, are suddenly struck down with a disease that has been for years insidiously creeping through the system and has finally asserted itself the master; and the doctor's carriage at the door from morn to night, the gathering of friends from far and near, the household hush, the crape on the knob, the hearse and a long line of black carriages, the cessation of the social round of dissipations for a fortnight, all tell the story of neglected health and the medical man's responsibility. It may be a delicate matter for a doctor to say "stop that lacing, and that baring of arms and necks, and that indulgence in improper food at unseasonable hours, and that over-exhaustion under the excitements of music and the whispered compliments of admirers—stop it or die;" it may be a terrible sacrifice to him to give up a paying patient in obedience to what he may deem the mawkish sentimentality of over anxious meddlers; it may be hard to abandon the role of a silent prescriber to ills which he had no hand in bringing about, and assume that other role of mentor to the fashionable, and subject himself to the charge of meddling with that which don't concern him; it may be fearfully disastrous to his prospects, especially if he be a young man just working into a lucrative practice; but when he considers that it is upon the decision he comes to his responsibility in this matter rests, and reflects upon the satisfaction that will ensue upon his washing his hands of any complicity in this wholesale degeneration of his race, surely he cannot hesitate. If there be those whose consciences are not troubled by any such reflections as these, and who are willing to endure the stigma which all sensible people must put upon a neglect to warn in time, then let them continue to heap up their wealth and their approbrium at the same time, until the period shall arrive when they will be held legally responsible for their omissions, as they are now held morally responsible.

